# A Staged Event Source Location Identification Scheme in Power Distribution Networks Under Extremely Low Observability

**DOI:** 10.3390/s25165169

**Published:** 2025-08-20

**Authors:** Xi Zhang, Jianyong Zheng, Fei Mei

**Affiliations:** 1 School of Cyber Science and Engineering, Southeast University, Nanjing 211189, China; jy_zheng@seu.edu.cn; 2 School of Electrical Engineering, Southeast University, Nanjing 210096, China; 3 College of Energy and Electrical Engineering, Hohai University, Nanjing 211100, China; meifei@hhu.edu.cn

**Keywords:** synchrophasor measurement, event source location identification, virtual event current injection, voltage measurement deviation, staged localization scheme

## Abstract

Recent advancements in synchrophasor measurement technologies have introduced an unprecedented level of visibility in power distribution networks (PDNs), providing a high-quality data foundation for the accurate perception of event source locations. However, the high cost and deployment expense pose a significant challenge in balancing system observability and event source location identification (ESLI) accuracy. In this paper, we propose a staged ESLI scheme based on voltage measurement deviation (VMD), which can achieve high-precision ESLI and event current calculations under extremely low-observability conditions, where the measurement devices are deployed only at the head substation and terminal buses. By setting an unknown event injection current and traversing each bus along the target feeder to derive the terminal bus voltage and its outgoing current, an ESLI model based on virtual event current injection (VCI) is constructed, which not only assists in the ESLI task but also confers the solving capability of the event current. Leveraging the event current calculation ability of the ESLI model, a VMD-based staged ESLI algorithm is developed, achieving an ordered and accurate search for the exact location of the event source in a goal-oriented manner. The effectiveness of the developed ESLI algorithm is evaluated on the IEEE 33-bus test system. Experimental results demonstrate that our VMD achieves high-precision ESLI and event current solving in PDNs under extremely low observability, significantly outperforming the state-of-the-art ESLI methods.

## 1. Introduction

Accurately locating the event source in power distribution networks (PDNs) is crucial for the rapid restoration of service to users and directly impacts the operational cost and reliability of the system [1,2]. In the past, ESLI methods for PDNs have generally been divided into two categories: traveling wave-based methods and impedance-based methods. Although traveling wave-based methods are highly accurate in transmission networks, their application in PDNs becomes extremely complex due to the presence of numerous laterals and the resulting reflected waves [3,4]. Moreover, these methods require expensive measurement devices with high sampling rates capable of detecting wavefronts propagating at near-light speeds. On the other hand, impedance-based methods estimate the event source location by calculating the impedance between the substation and the event point [5,6,7]. As a result, they are relatively simple to implement and cost-effective in practical engineering applications, but they often result in multiple possible solutions and exhibit a limited performance in accurately pinpointing the exact location of the event source.

With the continuous development of power communication and wide-area measurement technologies, data-driven ESLI methods based on phasor measurement units (PMUs) have emerged as a promising solution [8]. PMUs can capture time-synchronized three-phase voltage and current phasors from different buses in PDNs with a high reporting rate of 120 readings per second [9], which provides a solid data foundation for the ESLI task in PDNs. However, considering the high cost of PMUs, and the massive volume of monitoring data and high communication requirements associated with large-scale deployment, its widespread installation in the system is impractical [10]. Therefore, exploring highly accurate ESLI schemes using a limited number of PMUs holds a great practical significance.

To explore the ESLI scheme in PDNs under the low coverage rate of PMUs, Jamei et al. proposed an optimized fault section localization method, which can locate faults to adjacent bus clusters even under the condition of a sparse sensor deployment across the grid [11]. Yang et al. processed current vectors reconstructed via compressed sensing to determine all suspected fault sections and subsequently identified the actual fault section using voltage residuals and fault distances, achieving the improvement of the fault section localization accuracy [12]. Li et al. developed a faulted line localization method based on a convolutional neural network (CNN) classifier utilizing bus voltage measurements. Despite a significant reduction in system observability, this method is still able to locate the faulted line to a small neighborhood with a high probability [13]. Han et al. proposed a tuning-free fault feeder selection and section location method based on the synchronous Lissajous curve slope, addressing the poor generality and reliability of traditional high-impedance grounding fault location methods under complex fault scenarios [14]. Anguswamy et al. developed a novel method for fault detection, classification, and localization, which employs optimally placed distribution-level PMUs to track positive, negative, and zero-sequence components considering unique distribution network attributes [15]. Yildiz et al. proposed a strategic PMU deployment approach integrating a CNN, where the strategic placement of a limited number of PMUs can significantly enhance the success rate of the line outage detection [16]. However, although these studies effectively reduce the PMU coverage, they still require the installation of additional sensors in other buses within the system to assist in ESLI. Furthermore, they can only identify a broad region or a specific line as a potential event source candidate, without access to any precise location information along the line. As a result, the manual inspection of the specific line is still necessary to determine the exact event point. To achieve the exact location identification of event sources while ensuring economic feasibility in measurement device deployment, Farajollahi et al. proposed an ESLI method based on the forward–backward sweep (FBS), which requires only a very small number of PMUs installed at the substation and terminal buses to enable an accurate ESLI [17]. Due to its excellent localization performance under extremely low-observability conditions, FBS has been widely applied in the ESLI field [17,18,19]. However, it is only capable of localizing the event source to a specific system bus, without providing information about its exact location between two system buses. Moreover, FBS lacks the capability to calculate event currents and thus cannot provide further situational awareness of the event. To address the aforementioned issues, Sodin et al. improved the FBS-based ESLI method by introducing the concept of virtual buses, enabling it to have the ability to search for the exact location of the event source within a line [20]. However, when performing the localization task for laterals, this method relies on the assumption that the event impedance exhibits purely resistive characteristics to achieve the ESLI. Thus, it is only applicable to short-circuit fault events and fails to locate the event position along the lateral for event types lacking this characteristic, such as capacitor bank switching (CBS) and load switching (LS). Additionally, the method requires the deployment of smart meters (SMs) in all load buses to assist in observation. Buzo et al. proposed a voltage sag measurement (VSM)-based ESLI method applicable to PDNs under extremely low observability. By constructing a line impedance matrix and combining it with the VSM criterion, the method is capable of localizing the event to a specific point within a line [21]. However, it neglects the influence of all loads within the system, resulting in significant deficiencies in the event current calculation accuracy. This limitation explains why, even for single-line-to-ground (SLG) faults with relatively large event current magnitudes, the identification accuracy of the exact fault location reaches only 60% in the IEEE 33-bus test system. In addition, several studies have utilized PMUs to achieve the exact point localization of the event source in PDNs [22,23,24]. Nevertheless, these methods still require the deployment of a certain number of measurement devices in other buses within the system to assist in observation.

In summary, in addition to deploying measurement devices at the head substation and terminal buses, the vast majority of existing ESLI studies require the deployment of additional measurement equipment in other buses within the system to assist in localization. In addition, ESLI schemes that meet the extremely low-observability conditions exhibit significant limitations in both the exact point localization and event current calculation accuracy. To address the two major challenges, this paper proposes a VMD-based staged ESLI algorithm tailored for extremely low-observability PDNs. Firstly, by modeling the unknown event current and traversing all buses along the target feeder, an ESLI model based on virtual current injection (VCI) is constructed, which not only facilitates the ESLI task but also confers the capability of event current solving. Then, leveraging the event current calculation capability of the ESLI model, a VMD-based stage ESLI algorithm is developed, achieving a goal-oriented orderly search for the event source location. Finally, the ESLI performance and robustness of the VMD are tested based on the IEEE 33-bus test system, and the experimental results validate the effectiveness and superiority of the proposed algorithm.

## 2. Problem Statement

### 2.1. Accuracy Deficiencies in the Exact Location Identification of the Event Sources in PDNs Under Extremely Low Observability

High-precision ESLI solutions under extremely low-observability conditions can accurately perceive the abnormal state of the system while greatly reducing the equipment deployment cost and optimizing the economic operation of the system. At present, the advanced ESLI schemes demonstrating excellent localization performances under such constraints mainly include FBS [17,18,19] and the VSM [21]. FBS searches for the bus with the smallest nodal voltage difference within the system through the forward–backward sweep theory, and can achieve excellent ESLI effects. However, its ESLI target is only limited to system buses. When an event occurs at an exact point within a line, it can only identify the nearest system bus and fails to capture the exact location information. On the other hand, the VSM enables the perception of the exact location of the event source along a line by introducing fictitious buses. However, due to its neglect of current diversion effects of system loads, it introduces significant errors in the event current calculation, leading to a certain degree of deviation between the localization result and the actual event location. Thus, how to achieve the high-precision identification of the exact location of event sources in PDNs under extremely low-observability conditions to optimize the balance between the economic efficiency of the device deployment and the accuracy of the ESLI to the greatest extent remains an urgent and challenging research issue. 

### 2.2. Insufficient Event Current Calculation Accuracy of Existing ESLI Methods in PDNs Under Extremely Low Observability

Acquiring accurate event current data can not only assist the ESLI task on laterals under extremely low observability but can also enable further situational awareness and status assessments of the detailed event information. However, when measurement devices are only deployed at the head substation and terminal buses in PDNs, due to the low observability of the system operation status monitoring data and the complex multi-lateral architecture of the distribution network, it is extremely challenging to accurately calculate the event current. At present, ESLI research involving event current calculations in PDNs under extremely low observability is limited. The VSM [21] achieved the calculation of the event current and ESLI under extremely low observability for the first time by constructing the line impedance characteristic matrix and based on the VSM criterion of the terminal buses. However, this method ignores the influence of the load current diversion. Given that PDNs typically contain multiple load buses, this will lead to a significant deviation in the calculation accuracy of the event current. Furthermore, the method focuses on short-circuit faults characterized by large event current magnitudes, without discussing the universality applied to events with smaller current magnitudes, such as high-impedance fault (HIF) and power quality (PQ) events. For HIF and PQ events, obtaining accurate event currents can not only assist in their situational awareness but also provide a reliable data basis for optimizing dispatch and control strategies as well as enabling intelligent operation and maintenance. Thus, in extremely low-observability PDNs, improving the calculation accuracy of the event current in ESLI methods remains a challenge that urgently requires resolution.

## 3. ESLI Model Based on Virtual Event Current Injection

**Figure 1:**
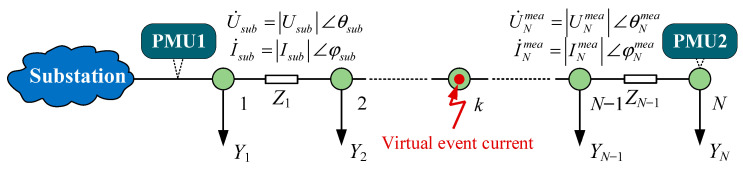
A representation of a distribution feeder when the VCI bus is set at bus *k*. Measurements are performed by two PMUs.

As illustrated in Figure 1, when an event is detected in the *N*-bus system, with only two PMUs deployed at the head substation and the terminal bus, we first assume system bus *k* as the event bus and model the event current as an unknown variable injected into the virtual event bus, i.e., the VCI bus. Then, based on the voltage and current measurements from PMU1, the forward sweep method is applied to derive the nodal voltage ${\dot{U}}_{N,k}$ and outgoing current ${\dot{I}}_{N,k}$ at the terminal bus *N*:

(1) $${\dot{U}}_{1,k}={\dot{U}}_{sub},\;{\dot{I}}_{1,k}={\dot{I}}_{sub}-Y_{1}{\dot{U}}_{1,k}\;\vdots \;{\dot{U}}_{k,k}={\dot{U}}_{k-1,k}-{\dot{I}}_{k-1,k}Z_{k-1},\;{\dot{I}}_{k,k}={\dot{I}}_{k-1,k}-Y_{k}{\dot{U}}_{k,k}+{\dot{I}}_{f,k}\;\vdots \;{\dot{I}}_{N-1,k}={\dot{I}}_{N-2,k}-Y_{N-1}{\dot{U}}_{N-1,k},\;{\dot{U}}_{N,k}={\dot{U}}_{N-1,k}-{\dot{I}}_{N-1,k}Z_{N-1}$$

where ${\dot{U}}_{sub}$ and ${\dot{I}}_{sub}$ represent the measured voltage and current phasors from PMU1 located at the substation, respectively; *Z_i_* represents the line impedance between bus *i* and *i* + 1; and *Y_i_* indicates the load lateral equivalent admittance of bus *i* and is considered as a pseudo-measurement. Note that since PMU2 is deployed at the terminal bus, when the VCI bus is set to bus *N*, it follows that ${\dot{I}}_{N,N}={\dot{I}}_{N-1,N}+{\dot{I}}_{f,N}$. Otherwise, ${\dot{I}}_{N,k}={\dot{I}}_{N-1,k}$.

By traversing all buses with the VCI bus, we can calculate two *N*-dimensional distributions of $U_{N}$ and $I_{N}$ with the unknown variable ${\dot{I}}_{f,k}$, thus constructing a mathematical model for ESLI in PDNs under extremely low observability. This model can be compared with the actual voltage and current measurements from PMU2 located at the terminal bus *N*, which not only provides reliable model support for the ESLI task, but also confers the capability to accurately calculate event currents, offering a crucial data foundation for proposing a staged ESLI scheme.

(2) $$U_{N}=\left[\begin{array}{c} {\dot{U}}_{N,1}\\ \vdots \\ {\dot{U}}_{N,k}\\ \vdots \\ {\dot{U}}_{N,N} \end{array}\right],\;I_{N}=\left[\begin{array}{c} {\dot{I}}_{N,1}\\ \vdots \\ {\dot{I}}_{N,k}\\ \vdots \\ {\dot{I}}_{N,N} \end{array}\right]$$

## 4. ESLI Algorithm Based on Voltage Measurement Deviation

### 4.1. Principles of the VMD-Based ESLI Algorithm

**Figure 2:**
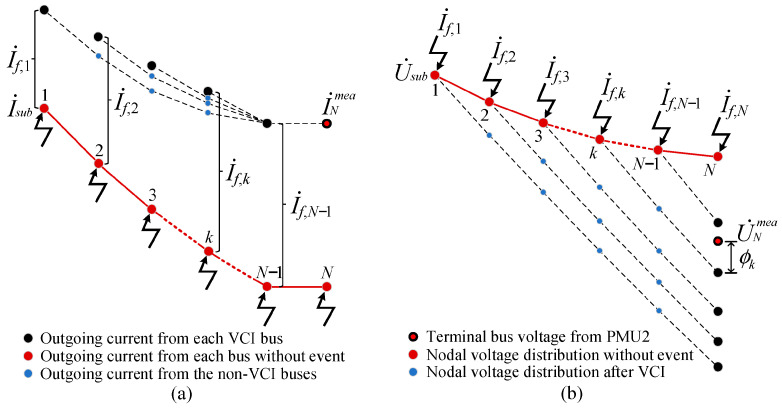
A schematic diagram of the VMD-based ESLI algorithm. (**a**) The VCI distribution solving based on the equivalence setting of the terminal outgoing current; (**b**) the VMD derived from the VCI distribution.

For the *N*-bus distribution feeder shown in Figure 1, since the voltage and current phasors at the terminal bus derived from the virtual event current injected at the actual event source location simultaneously satisfy the measurements from PMU2, we develop a voltage measurement deviation (VMD)-based localization criterion established on the equivalence setting of the terminal bus outgoing current, which can achieve an accurate ESLI while obtaining the precise event current phasor. To illustrate the underlying principle of the VMD, we explain it through theoretical derivation, and the principle schematic diagram is shown in Figure 2.

To more intuitively illustrate the impact of the VCI on the voltage/current distribution of the system, the nodal voltage and outgoing current (superscript s) of each bus derived from PMU1 measurements under the assumption of no event occurrence are taken as the benchmark parameters for analysis. First of all, we set bus *N* − 1 as the VCI bus and establish traversal settings to the upstream buses. When the VCI bus is *N* − 1, the terminal bus voltage ${\dot{U}}_{N,N-1}$ and the outgoing current ${\dot{I}}_{N,N-1}$ can be derived through (1) as

(3) $$\begin{array}{c} {\dot{I}}_{N,N-1}={\dot{I}}_{N,N-1}^{s}+{\dot{I}}_{f,N-1}\\ {\dot{U}}_{N,N-1}={\dot{U}}_{N,N-1}^{s}-{\dot{I}}_{f,N-1}\times Z_{N-1} \end{array}$$

Similarly, when bus *N* − 2 is set as the VCI bus, we can obtain the nodal voltage and outgoing current of bus *N* − 1:

(4) $$\begin{array}{c} {\dot{I}}_{N-2,N-2}={\dot{I}}_{N-2,N-2}^{s}+{\dot{I}}_{f,N-2}\\ {\dot{U}}_{N-1,N-2}={\dot{U}}_{N-1,N-2}^{s}-{\dot{I}}_{f,N-2}\times Z_{N-2} \end{array}$$

Building on this, another forward sweep can be performed to obtain the terminal bus voltage and current corresponding to the case where bus *N* − 2 is set as the VCI bus:

(5) $$\begin{array}{r} {\dot{I}}_{N,N-2}={\dot{I}}_{N-1,N-2}={\dot{I}}_{N-2,N-2}-Y_{N-1}\times {\dot{U}}_{N-1,N-2}=\\ {\dot{I}}_{f,N-2}+{\dot{I}}_{N-2,N-2}^{s}-Y_{N-1}\times ({\dot{U}}_{N-1,N-2}^{s}-{\dot{I}}_{f,N-2}\times Z_{N-2})=\\ {\dot{I}}_{N,N-2}^{s}+{\dot{I}}_{f,N-2}\times (1+Y_{N-1}Z_{N-2})\\ {\dot{U}}_{N,N-2}={\dot{U}}_{N-1,N-2}-{\dot{I}}_{N-1,N-2}\times Z_{N-1}={\dot{U}}_{N-1,N-2}^{s}-{\dot{I}}_{f,N-2}\times Z_{N-2}-\\ {}[{\dot{I}}_{N,N-2}^{s}+{\dot{I}}_{f,N-2}\times (1+Y_{N-1}Z_{N-2})]\times Z_{N-1}=\\ {\dot{U}}_{N,N-2}^{s}-{\dot{I}}_{f,N-2}\times [(1+Y_{N-1}Z_{N-1})Z_{N-2}+Z_{N-1}] \end{array}$$

Similarly, we can obtain the terminal bus voltage and current corresponding to the case where the VCI bus is set to bus *N* − 3:

(6) $$\begin{array}{r} {\dot{I}}_{N,N-3}={\dot{I}}_{N,N-3}^{s}+{\dot{I}}_{f,N-3}\times \{1+Y_{N-2}Z_{N-3}+Y_{N-1}\times \\ \left[Z_{N-3}+(1+Y_{N-2}Z_{N-3})Z_{N-2}]\right\}\\ {\dot{U}}_{N,N-3}={\dot{U}}_{N,N-3}^{s}-{\dot{I}}_{f,N-3}\times \{(1+Y_{N-1}Z_{N-1})\times [Z_{N-3}+\\ \left(1+Y_{N-2}Z_{N-3})Z_{N-2}]+(1+Y_{N-2}Z_{N-3})Z_{N-1}\right\} \end{array}$$

By an analogy, the expressions of the terminal bus voltage and its outgoing current corresponding to the injection of the virtual event current at each VCI bus can be summarized as Equations (7) and (8), thereby obtaining a more concise representation of the ESLI model based on VCI:

(7) $${\dot{U}}_{N,k}={\dot{U}}_{N,k}^{s}-{\dot{I}}_{f,k}\times \lambda (k)$$

(8) $${\dot{I}}_{N,k}={\dot{I}}_{N,k}^{s}+{\dot{I}}_{f,k}\times \varphi (k)$$

where the superscript *s* denotes the voltage/current derived from the PMU1 measurements and forward sweep under the assumption that bus *k* is not injected with the virtual event current; λ(k) and φ(k) represent the coefficients of the virtual event current in the expressions for the nodal voltage and outgoing current of terminal bus *N*, respectively. By observing the variations in coefficients λ(k) and φ(k) corresponding to each VCI bus, it can be concluded that as the location of the VCI bus shifts further upstream (i.e., as the bus index *k* decreases), both coefficients increase monotonically.

Based on the ESLI model and the measurements from PMU2, we propose a novel VMD-based ESLI criterion. The detailed procedure is as follows: Firstly, it is assumed that the outgoing current ${\dot{I}}_{N,k}$ (*k* = 1, 2, …, *N* − 1) at the terminal bus corresponding to each VCI bus is equal to the current ${\dot{I}}_{N}^{mea}$ measured by PMU2. Combined with the known ${\dot{I}}_{N,k}^{s}$ (it remains constant for any VCI bus *k* in the *N*-bus feeder shown in Figure 1) and φ(k), an *N* − 1-dimensional event current vector corresponding to buses 1 to *N* − 1 can be obtained:

(9) $${\dot{I}}_{f,k}=({\dot{I}}_{N,k}-{\dot{I}}_{N,k}^{s})/\varphi (k),\;k=1,2,\ldots ,N-1$$

Then, by substituting (9) into (7), the terminal bus voltage distribution corresponding to each VCI bus can be obtained.

(10) $${\dot{U}}_{N,k}={\dot{U}}_{N,k}^{s}-({\dot{I}}_{N,k}-{\dot{I}}_{N,k}^{s})\times \frac{\lambda (k)}{\varphi (k)}$$

Finally, the VMD value *ϕ_k_* corresponding to the VCI bus *k* is obtained by calculating the magnitude of the difference between ${\dot{U}}_{N,k}$ and ${\dot{U}}_{N}^{\mathrm{mea}}$ measured by PMU2:

(11) $$\phi_{k}=\left|{\dot{U}}_{N}^{mea}-{\dot{U}}_{N,k}\right|$$

When *ϕ_j_* approaches 0 (ideally equal to 0), the bus *j* is identified as the specific location of the event source; that is

(12) $$j=\arg_{k}\min\phi_{k}$$

To investigate the variation pattern of the terminal bus voltage ${\dot{U}}_{N,k}$ in (10) under the assumption that the outgoing currents at the terminal bus corresponding to all VCI buses are equal, we set ξ(k)=λ(k)/φ(k) and derive that the ξ(k) corresponding to the VCI bus starting from bus *N* − 1 is

(13) $$\xi (k)=\frac{\lambda (k)}{\varphi (k)}=\frac{\eta (k)}{1+Y_{N-1}\times \eta (k)}+Z_{N-1}$$

(14) $$\begin{array}{l} \eta (k)=0,\;k=N-1\\ \eta (k)=Z_{N-2},\;k=N-2\\ \eta (k)=Z_{N-2}+\frac{Z_{N-3}}{1+Y_{N-2}Z_{N-3}},\;k=N-3\\ \eta (k)=Z_{N-2}+\frac{\left(\frac{Z_{N-4}}{1+Y_{N-3}Z_{N-4}}+Z_{N-3}\right)}{1+Y_{N-2}\left(\frac{Z_{N-4}}{1+Y_{N-3}Z_{N-4}}+Z_{N-3}\right)},\;k=N-4 \end{array}$$

It can be seen from (14) that η(k) gradually increases as *k* decreases. Therefore, from (13), ξ(k) monotonically increases as *k* decreases. Furthermore, it can be known from (10) that as *k* decreases, the terminal bus voltage ${\dot{U}}_{N,k}$ monotonically decreases. That is, when the VCI bus index *k* gradually increases, the corresponding ${\dot{U}}_{N,k}$ is monotonically increasing, as shown in Figure 2b. Thus, only the ${\dot{U}}_{N,j}$ corresponding to the correct event bus *j* is equal to the ${\dot{U}}_{N}^{\mathrm{mea}}$ measured by PMU2, i.e., *ϕ_j_* = 0, thereby theoretically verifying the effectiveness of the proposed algorithm.

It should be noted that when the VCI bus is set to the terminal bus *N*, λ(N) and φ(N) in (7) and (8) are 0 and 1, respectively. When the actual event source is located at bus *N*, since ${\dot{U}}_{N}^{mea}={\dot{U}}_{N,N}$, then *ϕ_N_* = 0. For other VCI buses not located at bus *N*, we have $\phi_{k}=\left|\left({\dot{I}}_{N,k}-{\dot{I}}_{N,k}^{s})\times \xi (k\right)\right|$, and since (13) indicates that ξ(k) increases monotonically as *k* decreases, *ϕ_k_* likewise increases monotonically as *k* decreases. In other words, *ϕ_k_* attains its minimum at bus *N*. On the other hand, if the actual event source is located at a bus other than bus *N*, since ${\dot{U}}_{N}^{mea}\neq {\dot{U}}_{N,N}$, *ϕ_N_* will be greater than 0, thereby verifying that the VMD can also effectively and rapidly perceive whether the terminal bus *N* is the event bus.

### 4.2. Staged ESLI Scheme for PDNs with Laterals

**Figure 3:**
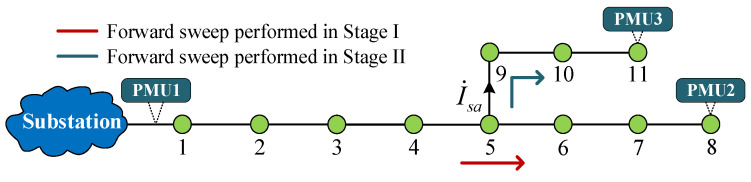
An 11-bus distribution network with a lateral.

Unlike single-feeder systems, the presence of laterals causes a current diversion on the main feeder, thereby altering the voltage and current distribution across the entire system and posing more severe challenges for the ESLI task. However, the VCI-based ESLI model, with its capability for precise event current calculations, can effectively eliminate the diversion effect of laterals and transform the complex multi-lateral system into a simplified single-feeder system for analysis, thereby enhancing the specificity of the ESLI. To facilitate understanding, we illustrate the process using an 11-bus PDN with a lateral, as shown in Figure 3. When an event is detected in the system, the localization process of the proposed ESLI algorithm is as follows:

(1) Stage I: ESLI on the Bus of the Main Feeder

Due to the VCI-based ESLI model’s comprehensive consideration of all buses in a single-feeder system as potential event source locations, it possesses the capability to calculate the phasor of the branch current when the event is assumed to occur at a branch bus. This enables the model to assist in completing the ESLI task on the target lateral when the actual event source is located there. Specifically, taking the 11-bus system shown in Figure 3 as an example, when the VCI bus is set as bus 1–4 or 6–8, since it is assumed that no event occurs on the lateral (bus 5–11), the branch current ${\dot{I}}_{sa}$ can be directly calculated through a backward sweep based on voltage and current measurements from PMU3. Consequently, the VCI-based ESLI model can be used to obtain the ${\dot{U}}_{N,k}$ and ${\dot{I}}_{N,k}$ (*k* ≠ 5) corresponding to these buses.

When the VCI bus is set as the branch bus 5, the branch current and virtual event current are treated as an overall unknown event current injected into that bus. On the one hand, this processing enables the accurate and rapid localization of the event source on the main feeder using the ESLI model and VMD criterion. On the other hand, if the event source is determined to be located at the branch bus, the solved event current ${\dot{I}}_{f,5}$ can be utilized to assist in the localization task for the target lateral.

(2) Stage II: ESLI on the Bus of Laterals

If Stage I locates the event to a branch bus on the main feeder, the obtained event current and the branch bus voltage derived based on the PMU1 measurements and forward sweep should be used, combined with the measurement results of PMU3, to further detect the event source location on the lateral using the VMD on the target lateral. It is worth noting that when the actual event source is located on the lateral, the nodal voltage and event current of the branch bus obtained through the VCI-based ESLI model, due to their sufficiently high accuracy, can serve as the PMU of the lateral head bus to assist in the ESLI on the lateral.

(3) Stage III: ESLI at the exact point between two buses

**Figure 4:**
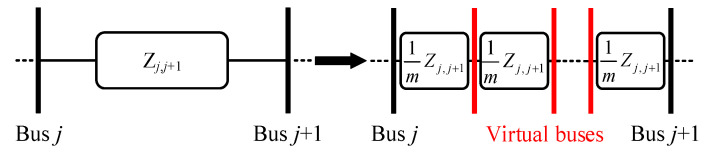
Schematic diagram of virtual buses.

When the event source is located at a certain point within a line, the search procedure of Stage I/II can only identify the system bus nearest to the actual location, without effectively pinpointing the exact point of the event source. To address this limitation, we introduce virtual bus technology to extend the ESLI capability of the VMD. As illustrated in Figure 4, assuming the line between buses *k* and *k* + 1 has an impedance of *Z_k_*_,*k*+1_, it is virtually divided into *m* equal parts, the number of which is determined according to the requirements of the task. A larger value of *m* corresponds to a higher ESLI accuracy.

Based on the line current between the event bus and its adjacent bus and their nodal voltage as determined in Stage I/II, the VMD can be used to pinpoint the exact location of the event source between two system buses by identifying the virtual bus index where the VMD value is extremely close to zero. The overall ESLI task execution process is detailed in Algorithm 1. It is worth noting that, unlike the exhaustive line analysis required between the event bus and all its adjacent buses in [17,18], the criterion used in the proposed ESLI algorithm is that the VMD equals zero; that is, the phasor difference between the calculated and the actual measured voltage at the terminal bus is zero. Moreover, since the VMD increases monotonically as the VCI bus deviates more from the actual event location, there is only one adjacent bus whose VMD phasor has both real and imaginary parts with opposite signs compared to those of the event bus. Leveraging this property allows for the rapid determination of which side of the event bus the event source is located.
**Algorithm 1** ESLI in PDNs with lateralsInput: PMU measurements and pseudo-measurements.Output: The location of the event source1:  /**/ Phase I: ESLI on the bus of main feeder** 2:  **if** *k *∈ the bus of main feeder Ω*_m_* is not a branch bus, **then** 3:   Use backward sweep to obtain the branch current based on the voltage/current measurements from the PMUs at the terminal bus of laterals. 4:  **else if**
*k *∈ Ω*_m_* is the branch bus, **then** 5:   Set its branch current equals as an overall unknown event current. 6:  **end if** 7:   Obtain the VCI and VMD distribution using (9) and (11). 8:   Obtain the event bus index by using (12). 9:  /**/ Phase II: ESLI on the bus of laterals** 10:   **for** ∀k ∈ the bus of target lateral Ω*_l_*, **do** 11:    Use VCI-based ESLI model to obtain terminal nodal voltage ${\dot{U}}_{N,k}$ and outgoing current ${\dot{I}}_{N,k}$. 12:    Obtain the VCI and VMD distribution using (9) and (11). 13:    Obtain the event bus index by using (12). 14:   **end for** 15:   /**/Phase III: ESLI at exact point between two buses** 16:   **for**
*k* = [*j*:1/*m*:*j* + 1] **do** 17:    Use VCI-based ESLI model to obtain terminal nodal voltage ${\dot{U}}_{j+1,k}$ and outgoing current ${\dot{I}}_{j+1,k}$. 18:    Obtain the VCI and VMD using (9) and (11). 19:   **end for** 20:   Obtain the event exact point by using (12).

## 5. Case Studies

**Figure 5:**
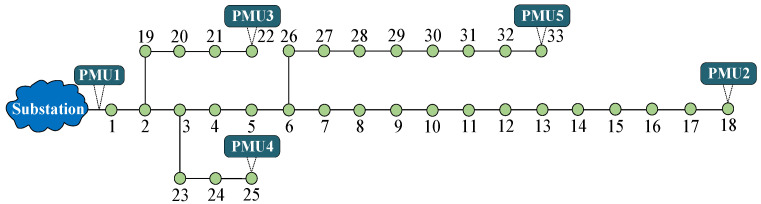
The schematic diagram of the IEEE 33-bus distribution network.

In this section, we construct the IEEE 33-bus test system on the MATLAB R2021b platform to evaluate the localization performance of the proposed VMD-based ESLI algorithm. The system topology is shown in Figure 5. To simulate the extremely low-observability scenario in PDNs, five PMUs are deployed at the head substation and the terminal buses. Each PMU reports synchronized voltage and current phasor measurements, with the essential measurement parameters summarized in Table 1. To ensure the proposed algorithm’s generalization capability across different event types, we consider both fault events, such as high-impedance faults (HIFs) and low-impedance faults (LIFs), and non-fault events, such as CBS and LS.

CBS and LS represent the most common reactive/active switching operations in the daily operation and automated control of distribution networks. These events occur frequently and cause notable transient disturbances in voltage and current waveforms. HIFs and LIFs, on the other hand, represent two typical fault scenarios with markedly different characteristics in terms of the fault current magnitude and duration. An HIF is characterized by a small current amplitude, susceptibility to background noise masking, and longer duration, while an LIF features a large current amplitude, a rapid rise rate, and distinct pulsed features, which can comprehensively cover the dynamic response characteristics from latent weak faults to typical short-circuit faults. Compared with other common event types in distribution networks (such as transformer switching, distributed power grid connection disturbances, reclosing operations, etc.), these processes can mostly be classified as active or reactive switching, and their transient characteristics are highly similar to LS and CBS. Meanwhile, higher-order fault forms such as arc grounding can also be regarded as an extension of HIFs. Therefore, the four selected event types are not only representative in terms of their occurrence frequency and engineering relevance but are also complementary across the two critical dimensions of active/reactive switching and weak/strong fault scenarios, which can fully verify the localization ability and generalization performance of the ESLI algorithm under both routine switching conditions and unexpected fault scenarios.

### 5.1. Scenario I: Capacitor Bank Switching Event

**Figure 6:**
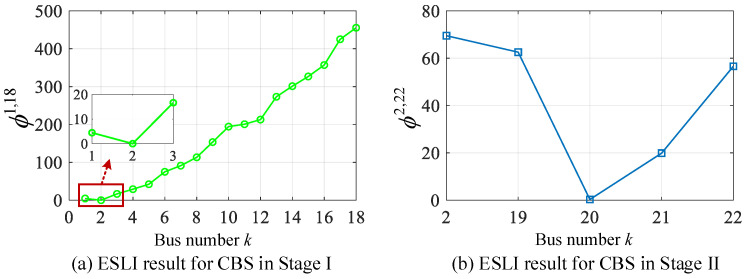
ESLI results for CBS on the main feeder in Stage I and the target lateral in Stage II.

Suppose a 700 kVAR capacitor is switched off at bus 20 located on lateral 1(bus 2–22). The proposed staged ESLI algorithm is used to obtain the VMD value *ϕ_k_* for each VCI bus of the main feeder and target lateral. Specifically, the VCI-based ESLI model and the VMD criterion are first used to perform a comprehensive sweep for all buses along the main feeder to obtain the VMD distribution corresponding to each VCI bus, as shown in Figure 6a. It can be observed that the VMD value at bus 2 is extremely close to zero. Thus, in the ESLI task of Stage I, CBS is located at branch bus 2, which indicates that the event source is located on lateral 1, and further location detection is required. Following the execution procedure of Stage II, the localization result on the target lateral is obtained, as illustrated in Figure 6b. As can be seen from Figure 6b, the VMD value *ϕ* at bus 20 approaches zero, leading to the identification of this system bus as the exact location of the event source, which is the correct event location. Furthermore, by examining the variation trends of VMD curves in Stage I/II, it can be seen that the VMD value increases monotonically as the VCI bus becomes more distant from the actual event location, which is consistent with the theoretical derivation presented in Section 4.1. Thus, the effectiveness and practicability of the VMD in the ESLI task for PDNs under extremely low observability are jointly verified from both theoretical and experimental aspects.

### 5.2. Scenario II: Load Switching Event

**Figure 7:**
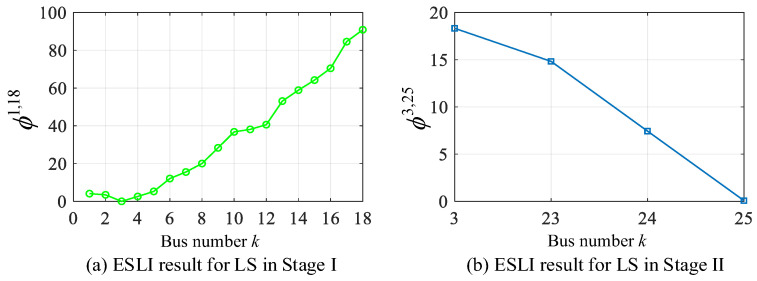
ESLI results of LS on the main feeder in Stage I and the target lateral in Stage II.

Suppose a three-phase 120 kW + 90 kVAR load switches on at bus 25 located on lateral 2 (bus 3–25). The proposed VMD-based algorithm is then employed to perform a staged ESLI task. The localization results for both Stage I and Stage II are shown in Figure 7. As observed from the figure, the event location is initially identified as branch bus 3 in the Stage I-ESLI on the main feeder and is subsequently pinpointed at bus 25 in the Stage II-ESLI on the target lateral, which corresponds to the actual event source location. This scenario further confirms the accuracy of our method.

### 5.3. Scenario III: High-Impedance Fault

**Figure 8:**
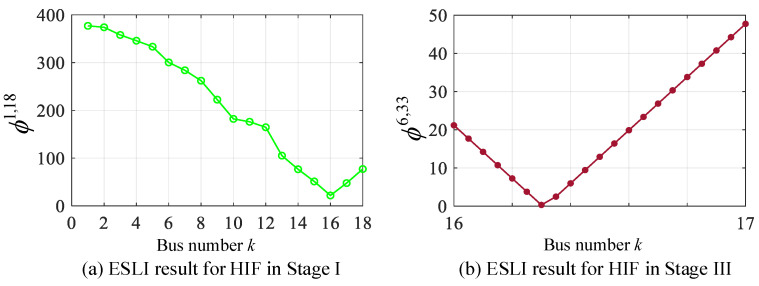
ESLI results for CBS on the main feeder in Stage I and the exact point in Stage III.

High-impedance faults may not interrupt service, but for safety reasons, they need to be accurately located to isolate the faulted area. Suppose a single-phase high-impedance fault (Phase B) with a 200 Ω fault resistance occurs in 30% of buses 16–17 located on the main feeder. The ESLI results of Stage I can be obtained by using the VMD, as shown in Figure 8a. It can be seen that the minimum value of *ϕ* points to bus 16. However, its value is significantly greater than zero, indicating that the exact location of the event is not at system bus 16 but within the section between it and its adjacent buses. To achieve the rapid determination of the specific section where the event source is located, the VMD phasor of bus 16 is calculated as −5.55 + 20.51i, while the VMD phasors of bus 15 and 17 are −9.53 + 50.31i and 17.22 − 44.52i, respectively. According to the adjacent section criterion discussed in Section 4.2, since the real and imaginary parts of the VMD phasors at bus 17 and bus 16 are opposite signs, it can rapidly determine the section where the event source is located as buses 16–17. Then, a total of 19 virtual buses are introduced into the line between bus 16 and bus 17 at a 5% interval, and the VMD is used to search for the exact location of the event source. The localization result of Stage III is shown in Figure 8b. It can be seen that the VMD accurately identifies the exact location of the HIF at 30% of buses 16–17.

### 5.4. Scenario IV: Low-Impedance Fault

**Figure 9:**
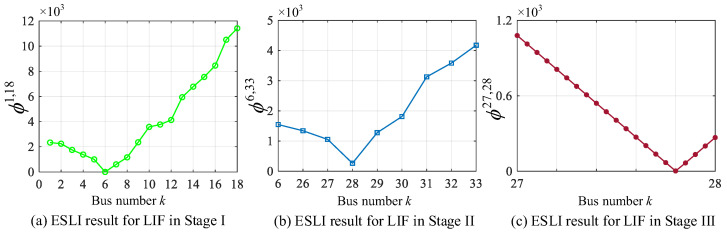
ESLI results for LIF on the main feeder in Stage I, the target lateral in Stage II, and the exact point in Stage III.

Low-impedance faults are typically accompanied by large event currents, posing significant risks to the safe and stable operation of power systems. Suppose a line-to-line short-circuit fault (Phase A/B) with a fault resistance of 5 Ω occurs in 80% of buses 27–28 located on lateral 3 (bus 6–33). Using the proposed VMD-based ESLI algorithm, the corresponding localization results for Stage I/II/III can be obtained, as shown in Figure 9a–c. During Stage I, the minimum *ϕ_k_* occurs at branch bus 6 and is close to zero, indicating that the event source is located on lateral 3. Through the detection of Stage II, *ϕ_k_* achieves the minimum value at bus 28. However, its value is not close to zero, suggesting that a further search is needed to identify the exact location within its vicinity. As shown in Figure 9c, the VMD can accurately locate the event source to the exact point located at 80% of the distance between bus 27 and bus 28.

### 5.5. Verification of Event Current Calculation Capability

Based on the four event scenarios described above, we install PMUs at each event location to monitor the precise phasor of the event current, which is used to compare it against the event current calculated by the proposed algorithm. The algorithm’s calculation accuracy is evaluated using the error absolute values of the real and imaginary components of the phasor, i.e., *δ*_real_/*δ*_imag_. The comparison results are summarized in Table 2. It can be seen that for the four different types of events, the overall average *δ*_real_ and *δ*_imag_ obtained using the VMD are only 0.037 and 0.098, respectively, indicating that it not only possesses the ability to precisely locate the event source, but also can obtain high-precision event current information, thereby verifying the effectiveness and superiority of the proposed algorithm in terms of its event current calculation capability.

### 5.6. Performance Comparison

In this section, we compare the proposed algorithm with three state-of-the-art ESLI methods for PDNs, namely FBS [17,18,19], improved FBS [20], and the VSM [21]. For the four aforementioned event scenarios, the ESLI results of various algorithms are summarized in Table 3. As shown in Table 3, FBS can accurately identify the event locations for CBS and LS. However, for the HIF and LIF, due to its inability to precisely localize events occurring within a line between two buses, it can only determine the system bus closest to the exact event point but cannot perceive its exact position. Compared with FBS, the improved FBS provides a precise localization function of the event source. However, its localization criterion on laterals is based on the assumption that the event impedance is purely resistive, identifying the event location by searching for the point where the imaginary part of the event impedance approaches zero. This implies that this method is only applicable to short-circuit fault events with purely resistive characteristics and is therefore unsuitable for PQ events such as CBS and LS. For these cases, the improved FBS can only identify the nearest branch bus on the main feeder. Furthermore, since a SM is deployed at each load bus to assist in observation, the setup does not strictly satisfy the extremely low-observability (ELO) condition. The VSM also has the function of precisely locating the event source by introducing fictitious buses. However, when constructing the impedance matrix, it neglects the influence of load impedances within the system, which can lead to significant errors in the event current calculation. In addition, the calculation of the VSM criterion uses the amplitude of the terminal nodal voltage rather than the phasor information, and the phase information has been proven to be one of the key factors affecting the ESLI accuracy [25]. As a result, although the VSM can accurately locate at least all four types of events to the sections adjacent to the actual event point, its disregard for the load current diversion and terminal voltage phase information prevents it from achieving the precise localization of the event source. In contrast, the proposed staged ESLI scheme based on the VMD leverages its high-precision event current solving capability to perform a goal-oriented orderly search for the exact event point within the system, which enables the accurate ESLI across various types of events. Meanwhile, combined with the VMD phasor real and imaginary part sign criterion that can rapidly determine the section where the event source is located, the specificity of the ESLI tasks has been enhanced.

### 5.7. Sensitive Analysis

Next, we use a Monte Carlo simulation to assess the impact of errors in parameters and measurements on the robustness of the proposed VMD. Within that, four scenarios with different levels of error and noise are performed using 500 simulations.

(1) Error in Line Parameters: The line inductance and resistance may deviate from their nominal values because of loading, aging, and weather conditions. Considering this uncertainty, the range of line parameters is generally set within ±5% of their nominal values [26]. Table 4 shows the overall results of the four aforementioned event scenarios when there are errors in the supposedly known line impedances. As we can see, for the line parameter error within 5% SD, the VMD achieves over a 92% accuracy in identifying the correct half section and exceeds an 82% accuracy in pinpointing the exact event point. Hence, the robustness of the proposed algorithm is confirmed for errors in line parameters.

(2) Errors in Pseudo-Measurements: Table 5 shows the ESLI accuracy for different levels of errors in pseudo-measurements. Even with errors with as high as a 100% SD, the VMD can still identify the correct half section of event sources in over 82% of random scenarios and have an exact point identification rate exceeding 74%.

(3) Errors in PMU Measurements: According to various field experiences and given the fact that the PMU has a very high precision with a typical accuracy of 0.01% in magnitude and 0.003° in angle [27], the proposed algorithm is tested at four different measurement error levels, with the results shown in Table 6. It can be seen that even with magnitude/angle errors reaching 0.1%/0.02°, the VMD can still identify the exact locations of event sources in over 98% of random scenarios. Thus, the robustness of the proposed ESLI algorithm is further confirmed. Note: To capture the dynamic evolution and characteristics of event signals in modern distribution networks, this study employs PMUs of Class M for voltage/current measurements, as they offer superior measurement accuracy, higher sampling rates, and broader frequency responses compared to Class P units [28].

### 5.8. Extension to Unbalanced Three-Phase Networks

**Figure 10:**
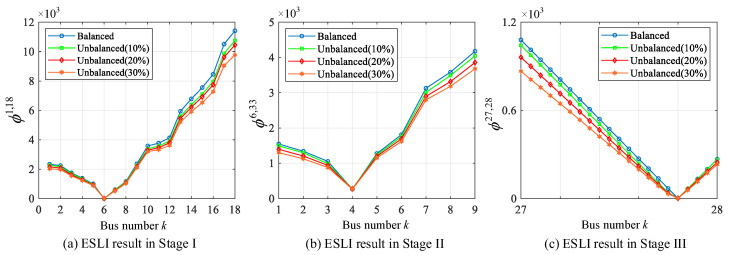
ESLI results for line-to-line short-circuit fault in different levels of unbalanced PDNs.

In this section, the proposed VMD is applied to unbalanced three-phase PDNs with asymmetric fault events. Specifically, the IEEE 33-bus test system is transformed into three levels of unbalanced three-phase networks by increasing and decreasing the load impedances of phase A and phase C by 10%, 20%, and 30%, respectively, while maintaining the loads present in phase B at 100%. Other parameters remain the same as those mentioned for the initial test system. We take the line-to-line short-circuit fault in Scenario IV as an example, which is the asymmetric fault event. Figure 10 shows the staged ESLI results of the proposed algorithm. It can be seen that under different levels of unbalance, the VMD distribution trajectories all exhibit noticeable deviations compared with the balanced case. This is mainly due to the sequence component propagation and the inter-phase coupling characteristics of the system under unbalanced conditions. Furthermore, as the level of imbalance increases, the VMD values of each VCI bus will further decrease. In other words, the distinctness of the VMD corresponding to the non-event VCI buses becomes weakened. However, even when the load unbalance level reaches 30%, the proposed algorithm can still pinpoint the event location clearly and accurately. Thus, we can conclude that the VMD can work well even for asymmetric fault events in unbalanced three-phase PDNs.

### 5.9. An Extended Application to the Larger Scale 69-Bus Distribution System

**Figure 11:**
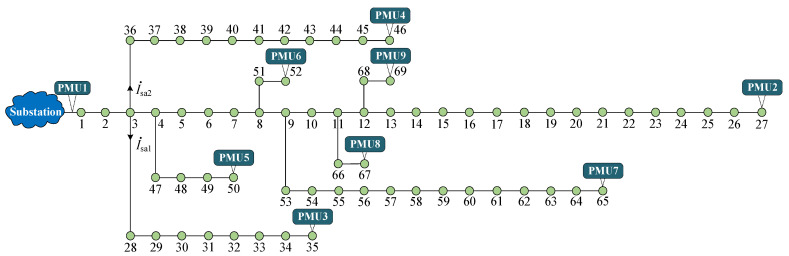
A schematic diagram of the IEEE 69-bus distribution network.

In order to investigate the performance of the proposed ESLI algorithm for a larger scale system, the simulation experiment is performed for the IEEE 69-bus and the 12.66 kV distribution network illustrated in Figure 11 [29,30]. We also define four different event scenarios to test the algorithm’s effectiveness. The detailed location and parameter information of four events are listed in Table 7.

**Figure 12:**
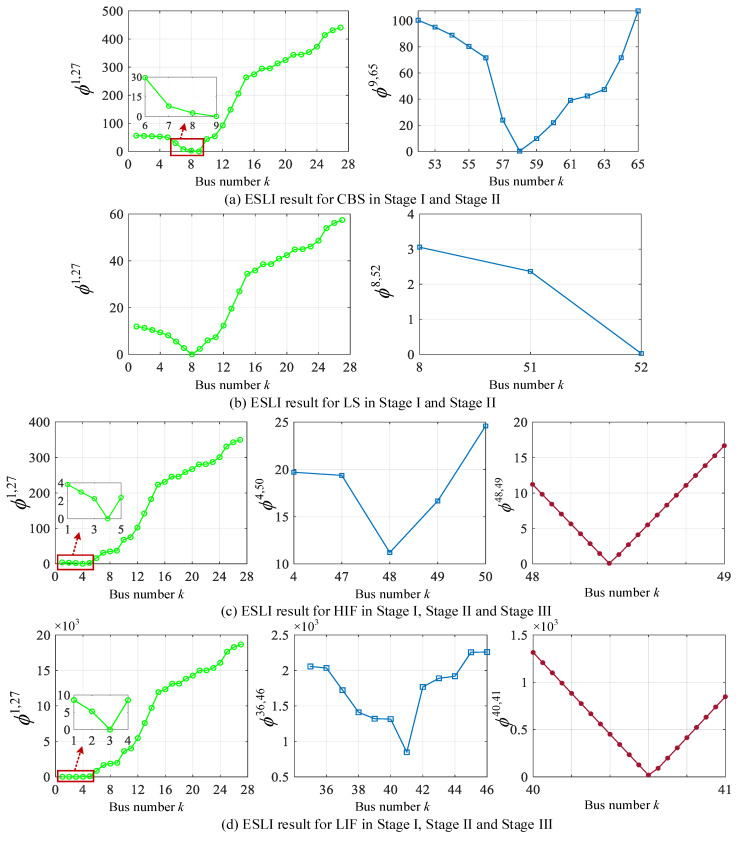
ESLI results of four event scenarios on the main feeder in Stage I, target lateral in Stage II, and exact point between two buses in Stage III.

The calculation results of locating four types of events using the VMD are shown in Figure 12. As can be seen, for the CBS and LS events, the VMD in Stage I locates them at bus 9 and bus 8 on the main feeder, respectively, and in Stage II it ultimately identifies their event locations as bus 58 and bus 52, which are the correct event locations. For the HIF event, the VMD locates it at bus 4 on the main feeder and bus 48 on the target lateral 3 (bus 4–50) in Stage I/II, respectively. Since the value of the VMD at bus 48 is not close to zero, it is necessary to perform the event exact point search task within its adjacent section. According to the calculation results of the VMD in Stage III, the position of the HIF is ultimately identified as 40% of buses 48–49, which is the correct event location. Furthermore, for the LIF event with a large event current, the VMD is used to locate it at bus 3, bus 41, and 60% of buses 40–41, respectively, in Stage I/II/III, which also corresponds to the actual event location. In conclusion, in the larger scale 69-bus system, the effectiveness and scalability of the proposed algorithm are validated.

**Figure 13:**
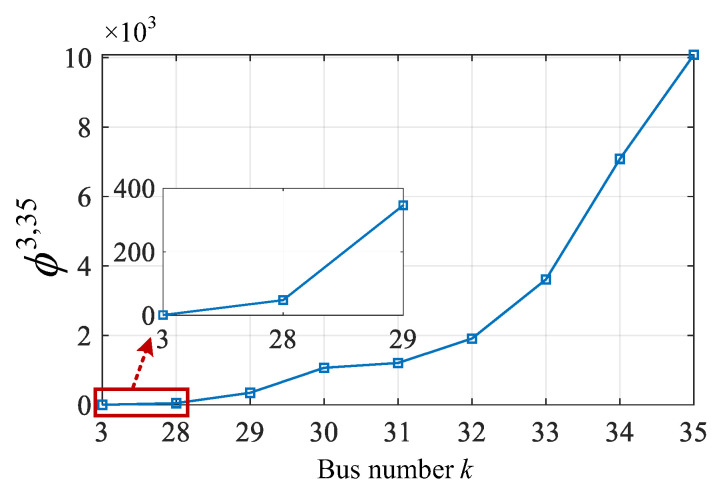
The ESLI result for the LIF on virtual event lateral 1 where the actual event is not located in Stage II.

It is worth noting that, unlike the 33-bus test system, there are two laterals at branch bus 3 in the 69-bus system, namely lateral 1 (bus 3–35) and lateral 2 (bus 3–46). When an event occurs on one of the two laterals, in Stage I, we regard the two laterals as a combined injection item. In other words, only an unknown event current is set to be injected into this bus. This ensures a rapid ESLI along the main feeder while also enabling the determination of the sum of the branch currents from the event lateral and the non-event lateral, given their parallel configuration. Although Stage I captures the combined injection current of the two laterals, it remains uncertain which specific lateral is associated with the event. Therefore, in Stage II a virtual event lateral is assigned to facilitate the localization analysis. In other words, one lateral is temporarily assumed to be the event lateral, while the other is considered unaffected. The branch current of the non-event lateral head bus can be computed based on the terminal PMU measurements and backward sweep. For instance, if lateral 2 is hypothesized as the event lateral, then using the voltage/current measurements from PMU3 and applying the backward sweep algorithm, the branch current ${\dot{I}}_{sa1}$ at the head of lateral 1 under non-event conditions can be derived. Since lateral 1 and lateral 2 are arranged in parallel, subtracting ${\dot{I}}_{sa1}$ from the combined injection current obtained in Stage I yields the branch current at the head bus of the virtual event lateral. Finally, combined with the nodal voltage of the branch bus derived in Stage I and the terminal PMU4 measurements, the event source exact location can be obtained by using the VMD. It should be noted that when the virtual event lateral is set to the lateral where the actual event source is not located, the localization result corresponds to its head bus. Taking the LIF event in Table 7 as an example, in Stage II, if lateral 2 is set as the virtual event lateral, the localization result is shown in Figure 12d. Conversely, when lateral 1 is assigned as the virtual event lateral, the ESLI result is shown in Figure 13. Thus, by combining the VMD distribution results with the two laterals, the actual event lateral and its exact location can be ultimately determined

In addition, following the validation approach described in Section 5.5, the event currents calculated using the VMD for four types of events in the 69-bus test system are compared against the actual measurements. A performance evaluation is conducted using the absolute error values of the real and imaginary components of the phasor, i.e., *δ*_real_/*δ*_imag_. The comparison results are presented in Table 8. It can be seen that, even within the larger scale 69-bus system, the overall average *δ*_real_ and *δ*_imag_ obtained using the VMD remain as low as 0.687 and 0.826, respectively, thereby confirming that the proposed algorithm continues to exhibit an excellent event current calculation capability in large-scale distribution networks.

## 6. Conclusions

To address the balance issue between the economic efficiency of the device deployment and the accuracy of the ESLI in PDNs, we propose a VMD-based precise localization algorithm, which achieves an accurate perception of the event source location and the high-precision calculation of the event current under extremely low observability. The main contributions of this paper are as follows:

(1) An ESLI model tailored for extremely low-observability PDN scenarios is developed, which is built upon the concept of VCI. By comparing the terminal bus voltage and its outgoing current corresponding to each VCI bus with actual terminal measurements from PMUs, the model not only assists in accomplishing the ESLI task but also confers the event current calculation capability.
(2) A staged ESLI algorithm based on the VMD is proposed. Leveraging the event current calculation capability of the VCI-based ESLI model and combining the equivalent setting of the outgoing current at the terminal bus with the VMD criterion, it can accurately capture the location information of the event source on the main feeder while obtaining the precise event current phasor. When the event source is located on a lateral, the event current on the branch bus calculated in the Stage I-ESLI task on the main feeder can assist in completing the ESLI task on the target lateral. This goal-oriented staged search scheme avoids the redundant detection of other laterals without events, reducing the computational complexity while enhancing the specificity of the ESLI process.
(3) By introducing virtual bus technology and using the VMD criterion, the accurate identification of the exact event point between two system buses under extremely low-observability PDN scenarios can be achieved. Experimental results demonstrate that the proposed VMD-based ESLI algorithm can accurately identify the exact locations of different types of events, including PQ and HIF events characterized by small event currents, as well as LIF events with large event currents. Meanwhile, the VMD exhibits a strong robustness against measurement and input parameter errors and maintains an excellent localization performance even in unbalanced three-phase systems.

In the future, we will focus on addressing two challenges. (1) ESLI efficiency optimization in PDNs: Although the VCI method provides high-precision ESLI model and event current calculation capabilities, it requires the repeated derivation and calculation of the terminal bus voltage and its outgoing current corresponding to each VCI bus based on the PMU1 measurement and forward sweep. Thus, the expression form of the localization model still requires further streamlining and optimization to enhance the ESLI efficiency and achieve the rapid awareness of event situations. (2) Development of high-precision ESLI schemes in PDNs after integrating distributed generations (DGs) under extremely low observability: With the advancement of the construction of the new power system, the penetration rate of DGs is constantly increasing, transforming the traditional single-source supply into a multi-source supply mode, making the power flow distribution in the system more complex and posing more arduous challenges to the ESLI task. Thus, in active PDNs without the deployment of additional measurement devices, the development of high-precision ESLI schemes suitable for extremely low-observability scenarios becomes a key technical issue for balancing ESLI accuracy and the economic feasibility of the measurement device deployment under the DG integration. (3) The enhancement of ESLI resilience against single-point PMU failures: Despite the proposed algorithm achieving high-precision ESLI under extremely low-observability conditions, it relies heavily on accurate voltage and current measurements from PMUs installed at the substation and terminal buses. The potential risks brought about by a single PMU failure may lead to the performance degradation of the ESLI method. Therefore, future research will focus on constructing an adaptive fusion mechanism based on topology awareness and data-driven approaches. By leveraging the limited information of the head and terminal buses and through means such as physical model reconstruction and historical event database learning, the state estimation and compensation of non-measured buses will be carried out on the basis of the relationship between topological links and power flows. Additionally, by incorporating the temporal characteristics of event propagation paths, a state evolution model within a virtual time window can be constructed to predict and repair potentially failed PMU data, thereby ensuring the continuity and fault tolerance of ESLI tasks.

## Figures and Tables

**Table 1:** The essential measurement parameters required by the proposed algorithm.

Parameters	Specific Requirements
Measurement resolution	120 frames per second (fps)
Measurement location	33-bus PDNs: Substation, bus 18/22/25/33
	69-bus PDNs: Substation, bus 27/35/46/50/52/65/67/69
Synchronization condition	GPS time synchronization error ≤ 1 μs

**Table 2:** A comparison between the event current calculated by the proposed algorithm and its actual measurement.

Data Source of Event Current	Types of Events			
	CBS	LS	HIF	LIF
Calculation	−0.205 − 30.259i	−0.919 − 6.412i	17.286 + 27.386i	943.611 + 144.207i
Measurement	−0.304 − 29.899i	−0.920 − 6.419i	17.299 + 27.362i	943.646 + 144.206i
Error (*δ*_real_/*δ*_imag_)	0.099/0.360	0.001/0.007	0.013/0.024	0.035/0.001

**Table 3:** ESLI performance comparison between VMD and three advanced ESLI methods in IEEE 33-bus system.

Event Type	FBS [17,18,19]			Improved FBS [20]			VSM [21]			Proposal		
	Section (Bus)	Exact Point	ELO Condition	Section (Bus)	Exact Point	ELO Condition	Section (Bus)	Exact = Point	ELO Condition	Section (Bus)	Exact Point	ELO Condition
CBS	20	20	Yes	2	2	No	18–20	19–20 (65%)	Yes	20	20	Yes
LS	25	25		3	3		24–25	24–25 (45%)		25	25	
HIF	16	16		16–17	16–17 (30%)		14–16	15–16 (80%)		16–17	16–17 (30%)	
LIF	28	28		27–29	27–28 (80%)		27–29	27–28 (70%)		27–28	27–28 (80%)	

**Table 4:** Sensitive analysis for error in line parameters.

Error in Line Parameters (SD)	Correct Section	Adjacent Section	Correct Half	Exact Point
2%	100%	0%	98.85%	93.70%
5%	100%	0%	92.05%	82.15%
8%	96.25%	3.75%	80.55%	61.90%
10%	94.55%	5.45%	68.65%	48.25%

**Table 5:** Sensitive analysis for error in pseudo-measurements.

Error in Power Injection (SD)	Correct Section	Adjacent Section	Correct Half	Exact Point
25%	100%	0%	100%	99.90%
50%	98.05%	1.95%	97.55%	92.85%
75%	94.45%	5.55%	90.80%	84.35%
100%	87.85%	11.95%	82.15%	74.60%

**Table 6:** Sensitive analysis for error in PMU measurements.

Error (Magnitude/Angle)	Correct Section	Adjacent Section	Correct Half	Exact Point
0.01%/0.003°	100%	0%	100%	100%
0.03%/0.006°	100%	0%	100%	100%
0.05%/0.01°	100%	0%	100%	99.75%
0.1%/0.02°	100%	0%	99.80%	98.25%

**Table 7:** Detailed information of four event scenarios in the IEEE 69-bus test system.

Event Type	Event Location		Parameter
	Phase	Exact Point	
CBS	a/b/c	Bus 58	700 kVAR
LS	a/b/c	Bus 52	120 kW + 90 kVA
HIF	b-g	40% of Bus 48–49	200 Ω
LIF	a-b-g	60% of Bus 40–41	5 Ω

**Table 8:** The comparison between the event current calculated by the proposed algorithm and its actual measurement in the 69-bus test system.

Data Source of Event Current	Types of Events			
	CBS	LS	HIF	LIF
Calculation	0.319 − 30.124i	−0.770 − 6.635i	18.346 + 31.468i	1874.779 + 500.285i
Measurement	0.763 − 29.436i	−0.771 − 6.644i	18.349 + 31.462i	1877.077 + 502.887i
Error (*δ*_real_/*δ*_imag_)	0.444/0.688	0.001/0.009	0.003/0.006	2.298/2.602

## Data Availability

The datasets used and/or analyzed during the current study are available from the first author upon reasonable request (first author E-mail address: 230229220@seu.edu.cn).
